# Antibiotic prophylaxis in emergency cholecystectomy for mild to moderate acute cholecystitis: a systematic review and meta-analysis of randomized controlled trials

**DOI:** 10.1186/s13741-024-00441-4

**Published:** 2024-08-09

**Authors:** Mohamed Hamouda Elkasaby, Hesham Elsayed, Dilawer Chofan Charo, Mohamed Abdalla Rashed, Omar Elkoumi, Islam Mohsen Elhaddad, Ahmed Gadallah, Alaa Ramadan

**Affiliations:** 1 Faculty of Medicine, Al-Azhar University, Cairo, Egypt; 2 Faculty of Medicine, Tanta University, Tanta, Egypt; 3 General Surgery Department, Ministry of Health, Latakia, Syria; 4 Faculty of Medicine, Suez University, Suez, Egypt; 5 Faculty of Medicine, Al-Azhar University, Damietta, Egypt; 6 Faculty of Medicine, Ain Shams University, Cairo, Egypt; 7 Faculty of Medicine, South Valley University, Qena, Egypt; 8 Medical Research Group of Egypt (MRGE), Cairo, Egypt

**Keywords:** Acute cholecystitis, AC, Antibiotics, Emergency cholecystectomy

## Abstract

**Background:**

Emergency cholecystectomy is the mainstay in treating acute cholecystitis (AC). In actual practice, perioperative prophylactic antibiotics are used to prevent postoperative infectious complications (PIC), but their effectiveness lacks evidence. We aim to investigate the efficacy of prophylactic antibiotics in emergency cholecystectomy.

**Methods:**

We searched PubMed, Embase, Cochrane CENTRAL, Web of Science (WOS), and Scopus up to June 14, 2023. We included randomized controlled trials (RCTs) that involved patients diagnosed with mild to moderate AC according to Tokyo guidelines who were undergoing emergency cholecystectomy and were administered preoperative and/or postoperative antibiotics as an intervention group and compared to a placebo group. For dichotomous data, we applied the risk ratio (RR) and the 95% confidence interval (CI), while for continuous data, we used the mean difference (MD) and 95% CI.

**Results:**

We included seven RCTs encompassing a collective sample size of 1747 patients. Our analysis showed no significant differences regarding total PIC (RR = 0.84 with 95% CI (0.63, 1.12), *P* = 0.23), surgical site infection (RR = 0.79 with 95% CI (0.56, 1.12), *P* = 0.19), distant infections (RR = 1.01 with 95% CI (0.55, 1.88), *P* = 0.97), non-infectious complications (RR = 0.84 with 95% CI (0.64, 1.11), *P* = 0.22), mortality (RR = 0.34 with 95% CI (0.04, 3.23), *P* = 0.35), and readmission (RR = 0.69 with 95% CI (0.43, 1.11),* P* = 0.13).

**Conclusion:**

Perioperative antibiotics in patients with mild to moderate acute cholecystitis did not show a significant reduction of postoperative infectious complications after emergency cholecystectomy. (PROSPERO registration number: CRD42023438755).

**Supplementary Information:**

The online version contains supplementary material available at 10.1186/s13741-024-00441-4.

## Introduction

Acute cholecystitis (AC) is an inflammatory disease of the gallbladder caused by gallstone obstruction of the cystic duct in 90% to 95% of cases, while acute acalculous cholecystitis accounts for 5% to 10% (Indar and Beckingham 2002). When the cystic duct is blocked, it causes high pressure in the gallbladder. This, combined with bile concentrated with cholesterol, starts an immediate inflammatory reaction (Gallaher and Charles 2022). Roughly 20% of people with AC also develop bacterial infections from enteric organisms like *E. coli*, *Klebsiella*, and *Streptococcus faecalis (*Kaplan et al. 2021*).*

AC is responsible for 20% of all cholecystectomy operations and is the third cause of all emergency admissions to surgical wards. Moreover, it accounts for 3% to 5% of hospitalizations worldwide (Payen et al. 2011). In the United States, approximately 10% of adults suffer from cholelithiasis, and the most common complication is acute calculous cholecystitis. The costs associated with this condition exceed $6.3 billion annually, making it a significant burden. Over the past 30 years, there has been a more than 20% increase in cases, further highlighting the severity of the issue (Shaffer 2005; Kimura et al. 2007).

Emergency cholecystectomy within three days of diagnosis is the mainstay in treating AC (Gallaher and Charles 2022). The complication rate after emergency cholecystectomy varies from 15 to 30%, with postoperative infectious complications (PIC) being the most common (Cao et al. 2015). In actual practice, perioperative antibiotics are used to prevent complications, but their effectiveness lacks evidence (Gomi et al. 2018).

In elective cholecystectomy, there are recommendations for the use of prophylactic preoperative antibiotics to reduce the incidence of PIC. However, in emergency cholecystectomy, the evidence is still scarce (Yan et al. 2011; Sharma et al. 2010; Vohra et al. 2017; Gomez-Ospina et al. 2018). Although the Surgical Infection Society and the Tokyo Guidelines recommend the use of antibiotic prophylaxis in emergency cholecystectomy, these recommendations are not supported with sufficient evidence (Gomi et al. 2018; Mazuski et al. 2017). A randomized trial by Regimbeau et al. (2014) concluded that there is no significant difference in PIC with or without antibiotics (Jaafar et al. 2020).

We hypothesized that administering perioperative prophylactic antibiotics in emergency cholecystectomy in patients with AC may be ineffective due to the inflammatory rather than infectious nature of the condition. In this line, we conducted this study to test our hypothesis by gathering all published randomized controlled trials (RCTs) in this meta-analysis.

## Methods

We conducted our systematic review and meta-analysis following the Cochrane Handbook for Systematic Reviews of Intervention (Shea et al. 2007) and the AMSTAR-2 (Assessing the Methodological Quality of Systematic Reviews 2) Guidelines (Shea et al. 2007). We strictly followed the PRISMA (Preferred Reporting Items for Systematic Reviews and Meta-analyses) guideline (Moher et al. 2009) when reporting this meta-analysis. We registered the protocol of this study in the International Prospective Register of Systematic Reviews (PROSPERO) prior to conducting our study (registration number: CRD42023438755).

### Search strategy

We searched PubMed, Embase, Cochrane CENTRAL, Web of Science (WOS), and Scopus up to June 14, 2023. We used keywords of cholecystectomy, acute, emergency, and antibiotic to find relevant studies comparing perioperative antibiotic administration with placebo in patients undergoing emergency cholecystectomy. No filters were used when searching databases. The full strategy is summarized in Supplementary Table S1.

### Eligibility criteria

We included RCTs that involved patients diagnosed with mild to moderate AC according to Tokyo guidelines (Gomi et al. 2018) who were undergoing emergency cholecystectomy and were administered preoperative and/or postoperative antibiotics as an intervention group and compared to a placebo group. The main outcome of interest was the occurrence of PIC. Observational studies, case reports, case series, book chapters, research using non-human participants, studies not presented in the English language, and conference abstracts were not included.

### Studies selection

To eliminate duplicates, we utilized the EndNote Reference Library (EndNote X9 Version, Clarivate, Philadelphia, PA, USA). Next, we uploaded the studies to the Rayyan website (Ouzzani et al. 2016) for screening, which was performed by two teams, each consisting of two members. With the blinding feature enabled, we conducted title and abstract screening. Afterwards, we proceeded to conduct full-text screening for the included studies before finalizing our selection. The decision for each study was made independently by at least two authors, with another member responsible for reviewing any conflicts.

### Quality assessment

To assess the quality of the RCTs included in our study, we used the Cochrane Collaboration Risk of Bias Assessment Tool 2 (ROB2) (2023), which evaluates the following domains: randomization, deviations from intended interventions, missing outcome data, measurement of the outcome, selection of the reported result, and overall bias. We classified the outcome of the process as low, unclear, or high risk. Two reviewers conducted the risk of bias assessment independently. In case of any discrepancies, we resolved them by team discussion.

### Data extraction

The data extracted were as follows: (1) a summary of included studies, e.g., title, study design, country, duration, inclusion and exclusion criteria, antibiotic name, dosage, route of administration, and follow-up, and (2) baseline characteristics of the enrolled patients, e.g., sample size, age, sex, and body mass index (BMI). We extracted data on these outcomes: total PIC, surgical site infection (SSI), superficial SSI, deep SSI, organ and/or space SSI, postoperative distant infections, pneumonia, urinary tract infection (UTI), mortality, readmission, length of hospital stay, operation time, and total postoperative non-infectious complications.

### Statistical analysis

To conduct our analysis, we utilized the Review Manager software (RevMan for Windows, version 5.4, the Cochrane Collaboration, 2020). For dichotomous data, we applied the risk ratio (RR) and the 95% confidence interval (CI), while for continuous data, we used the mean difference (MD) and 95% CI. Statistical significance was considered if the *p* value was less than 5%. To assess statistical heterogeneity among the pooled results, we used the I-squared test (*I*^2^). If the *I*^2^ statistic exceeded 50% or the corresponding *p*-value was less than 0.1, the pooled results were considered heterogeneous, and we used the random effect model. Otherwise, we utilized the fixed-effect model. We conducted a subgroup analysis of antibiotic administration timing, grouping them as preoperative or postoperative. We conducted a sensitivity analysis using the leave-one-out model to account for significant heterogeneity.

### Adherence to the registered protocol

In the protocol registered in the PROSPERO register, it was not planned to conduct a subgroup analysis. We decided to conduct the subgroup analysis on the timing of antibiotic administration during the data extraction phase. This decision was made to investigate whether different timings might yield varied outcomes. Initially, we were unsure if the available data would support this analysis, which is why it was not included in the original study protocol registered on PROSPERO.

### Clarity of the evidence

Two researchers evaluated the certainty of evidence using the Grading of Recommendations Assessment, Development and Evaluation (GRADE) (2023) through the GRADE Pro online website tool (GRADEpro 2023). We assessed the quality of the evidence and the confidence in the effect estimates based on study design, risk of bias, inconsistency, indirectness, imprecision, and others. The scale was stratified as follows: high quality, which means no further research is needed and unlikely to change the confidence of the effects estimations; moderate quality, which means that further studies may affect the confidence of the effects estimation; low quality, which means further research is likely to have a crucial impact on the confidence of the effects estimation and may change the estimation; and very low quality, which means that we cannot be certain about this estimation (Table 1).

**Table 1:** Certainty of evidence according to the grading of recommendations assessment, development, and evaluation (GRADE) scale

Certainty assessment							№ of patients		Effect		Certainty	Importance
№ of studies	Study design	Risk of bias	Inconsistency	Indirectness	Imprecision	Other considerations	[Antibiotics]	[Placebo]	Relative (95% CI)	Absolute (95% CI)		
**Total postoperative infectious complications**												
7	Randomized trials	Serious^a^	Not serious	Not serious	Not serious^b^	None	78/866 (9.0%)	95/881 (10.8%)	**RR 0.84** (0.63 to 1.12)	**17 fewer per 1000** (from 40 fewer to 13 more)	⨁⨁⨁◯ Moderate	CRITICAL
**Total surgical site infections**												
6	Randomized trials	Serious^a^	Not serious	Not serious	Not serious^b^	None	2/651 (0.3%)	8/655 (1.2%)	**RR 0.79** (0.56 to 1.12)	**3 fewer per 1000** (from 5 fewer to 1 more)	⨁⨁⨁◯ Moderate	CRITICAL
**Total distant infections**												
5	Randomized trials	Serious^a^	Not serious	Not serious	Not serious^b^	None	21/820 (2.6%)	20/837 (2.4%)	**RR 1.01** (0.55 to 1.88)	**0 fewer per 1000** (from 11 fewer to 21 more)	⨁⨁⨁◯ Moderate	CRITICAL
**Total postoperative non-infectious complications**												
6	Randomized trials	Serious^a^	Not serious	Not serious	Not serious^b^	None	78/824 (9.5%)	94/833 (11.3%)	**RR 0.84** (0.64 to 1.11)	**18 fewer per 1000** (from 41 fewer to 12 more)	⨁⨁⨁◯ Moderate	IMPORTANT
**Mortality**												
5	Randomized trials	Serious^a^	Not serious	Not serious	Very serious^d^	None	0/699 (0.0%)	2/711 (0.3%)	**RR 0.34** (0.04 to 3.23)	**2 fewer per 1000** (from 3 fewer to 6 more)	⨁◯◯◯ Very low	CRITICAL
**Readmission**												
5	Randomized trials	Serious^a^	Not serious	Not serious	Serious^c^	None	28/731 (3.8%)	41/742 (5.5%)	**RR 0.69** (0.43 to 1.11)	**17 fewer per 1000** (from 31 fewer to 6 more)	⨁⨁◯◯ Low	IMPORTANT
**Length of hospital stay**												
5	Randomized trials	Serious^a^	Serious^e^	Not serious	Not serious	None	617	626	-	MD **0.89 Day higher** (0.14 lower to 1.92 higher)	⨁⨁◯◯ Low	IMPORTANT

*Abbreviations*: *CI* confidence interval, *MD* mean difference, *RR* risk ratio

^a^Some of the included studies have a high risk of bias

^b^The optimal information size criterion is met, and the 95% CI overlaps no effect (i.e., CI includes RR of 1.0), but CI excludes important benefits and harm

^c^The optimal information size criterion is met, the 95% CI overlaps no effect (i.e., CI includes RR of 1.0), and the CI fails to exclude important benefits

^d^The optimal information size criterion is met, the 95% CI overlaps no effect (i.e., CI includes RR of 1.0), and the CI fails to exclude important benefits and harm

^e^There was a significant heterogeneity (*I*^2^ = 98%)

## Results

### Search literature results

**Fig. 1:**
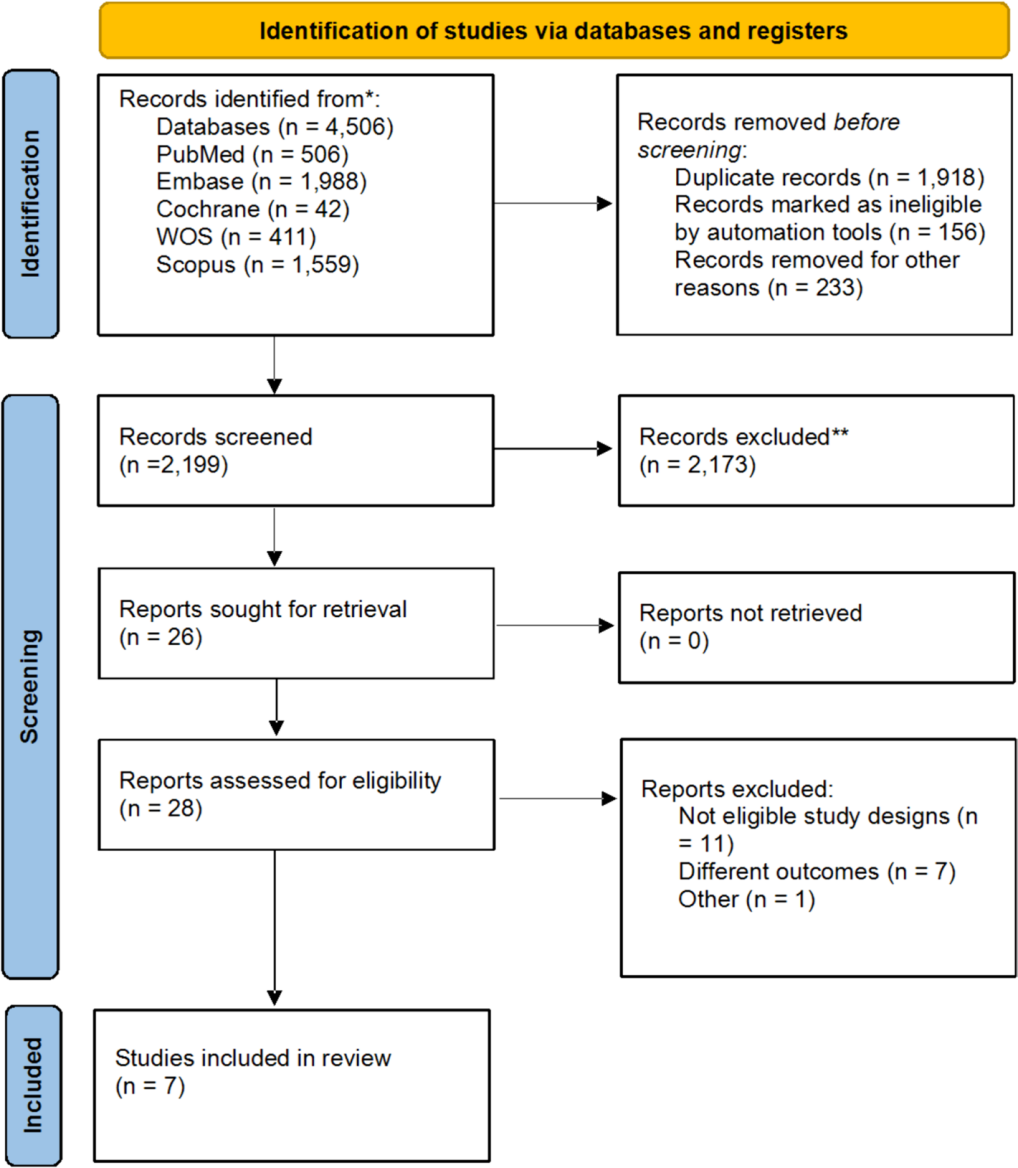
PRISMA flow diagram of the included studies

Our search resulted in a total of 4506 records; after the duplicates were removed, 1918 records entered the process of title and abstract screening. Twenty-six articles were eligible for the full-text screening, and finally, seven studies (Jaafar et al. 2020; Regimbeau et al. 2014; Braak et al. 2022; Park et al. 2023; Kim et al. 2017; Loozen et al. 2017; Santibañes et al. 2018) were available to enter our meta-analysis. The selection process of the included studies is shown in Fig. 1.

### Characteristics of the included studies

Out of the seven studies, two were conducted in South Korea (Park et al. 2023; Kim et al. 2017), two in the Netherlands (Braak et al. 2022; Loozen et al. 2017), and one in Argentina (Santibañes et al. 2018), France (Regimbeau et al. 2014), and Sweden (Jaafar et al. 2020). These studies encompassed a time frame spanning from 2009 to 2021 and had a collective sample size of 1747 patients. Of these patients, 866 were administered antibiotics, while the remaining 881 patients constituted the control group. All included patients had mild to moderate AC. Laparoscopic cholecystectomy (LC) was the surgical technique employed in all of the studies included in the analysis. Regimbeau et al. (2014) utilized open cholecystectomy in 6.8% (14 patients) in the antibiotic group and 5.3% (11 patients) in the control group. Jaafar et al. (2020) included four patients who were initially scheduled to have LC; nevertheless, as a result of technical concerns, the surgeon decided to do an open surgical procedure instead. The summary of included studies and baseline characteristics are reported in Tables 2, and 3.

**Table 2:** Summary of the included studies

Study ID		Braak et al.^2022^	Jaafar et al. 2020	Park et al. 2023	Kim et al. 2017	Loozen et al. 2017	Regimbeau et al. 2014	Santibañes et al. 2018
**Study design**		Randomized, controlled, open-label, clinical trial	Double-blinded, placebo-controlled, randomized study	Double-blinded, placebo-controlled, randomized study	Randomized controlled trial	Randomized controlled, open, parallel-group, noninferiority trial	Open-label, noninferiority, randomized clinical trial	Single-center, randomized, controlled, double-blinded trial
**Country**		The Netherlands	Sweden	South Korea	South Korea	The Netherlands	France	Argentina
**Duration**		From March 2016 to February 2020	From 14 December 2009 to 4 April 2017	From March 2019 to October 2021	From August 2015 to April 2016	From April 2012 to October 2014	From May 2010 to August 2012	From February 2014 to March 2017
**Inclusion criteria**		All adult patients presenting with ACC, in whom the intention was to perform immediate LC, were assessed for eligibility	Clinical and radiological signs of AC grades I and II suitable for acute LC and age ≥ 18 years	AC grade I and IIa patients, according to Tokyo Guidelines 2018	Patients with mild or moderate ACC undergoing laparoscopic cholecystectomy	Adult patients suffering from mild ACC with an Acute Physiology and Chronic Health Evaluation (APACHE) II score of 6 or lower	Patients aged 18 years or older with mild (grade I) or moderate (grade II) ACC (as defined by the Tokyo consensus meeting)	Diagnosis of mild or moderate ACC men and nonpregnant, nonlactating women between 18 and 85 years of age who undergo early LC
**Exclusion criteria**		Patients who presented with severe cholecystitis, received antibiotics, acalculous cholecystitis, already receiving or needing antibiotic treatment for a concomitant infection or sepsis, proven allergy to cefazolin, pregnancy, or an indication for ERCP on admission	Ongoing septicemia, pregnancy, bile duct obstruction, contraindication to LC, treatment with antibiotic drugs within 24 h, and symptom duration longer than 5 days	Immunodeficiency, concurrent operation on other organs, suspicion of malignancy, history of previous upper abdominal surgery, suspicion of a hollow organ injury, exploration of the common bile duct or conversion to laparotomy during the operation	If the boundary of the GB was already dissolved owing to severe inflammatory changes in the wall structure, as in the case of GB perforation. Any evidence of bile peritonitis during the operation. Immunodeficiency, concurrent operation on other organs, suspicion of malignancy, history of previous upper abdominal surgery, suspicion of hollow organ injury, or exploration of the common bile duct or conversion to laparotomy during the operation	Age < 18 years, antibiotics before diagnosis of cholecystitis, known allergy to cefuroxime or metronidazole, pregnancy, indication for ERCP on admission, abnormal liver test results with suspicion of acute cholangitis	Grade III severe ACC (with an indication of percutaneous transhepatic biliary drainage or required emergency cholecystectomy for septic shock, complaints lasting form or more than 5 days, common bile duct stones discovered at the time of surgery, cholangitis, biliary peritonitis, acute pancreatitis, cirrhosis, suspected biliary cancer, β-lactam allergy, and pregnant or breastfeeding	Hypersensitivity to amoxicillin or clavulanic acid or lactose (used in placebo); severe ACC; moderate ACC associated with liver and/or gallbladder abscesses, cholangitis, or bile peritonitis, intraoperative findings such as liver cancer, liver metastases, common bile duct stones, or gallbladder carcinoma, conversion to laparotomy, previous treatment with antibiotics for > five days, active oncologic diseases, AIDS, and transplant patients
**Antibiotic**	**Name**	First-generation cephalosporin (cefazolin)	Piperacillin/tazobactam	First-generation cephalosporin (cefazolin)	Second-generation cephalosporin (cefoxitin sodium)	Cefuroxime and metronidazole	Amoxicillin/clavulanic acid	Amoxicillin/clavulanic acid
	**Route of administration**	Intravenously	Intravenously	Intravenously	Intravenously	Intravenously	Intravenously	Orally
	**Dosage**	Single dose, 2 g, 15–30 min before surgery	4 g. As the time between inclusion and the procedure varied, infusions were given over periods varying from less than an hour to 72 h	Empirical antibiotics, 1 g of first-generation cephalosporin (cefazolin)	All patients received preoperative antibiotics with 1.0 g of second-generation cephalosporin (cefoxitin sodium) three times a day intravenously from the time of diagnosis of AC and received a single dose of antibiotics 30 min before surgery. The same antibiotic was routinely given once more during the operation. After surgery, patients were given either the placebo (group A) or postoperative antibiotics (cefoxitin) (group B). In group B, all patients received 1.0 g of cefoxitin three times a day postoperatively and then switched to oral pills (cefaclor, 250 mg per pill, two times a day)	Once included, patients received a single prophylactic dose of antibiotics 15–30 min before surgery (cefazolin 2000 mg intravenously). The antibiotic group was admitted for 3 days after surgery to receive intravenous cefuroxime 750 mg and metronidazole 500 mg three times daily	The treatment group received the same antibiotic regimen three times daily for 5 days. Patients who were not yet eating received 2 flasks of 1 g/200 mg intravenously, and those who could eat received 2 pills of 1 g each. Patients discharged within 5 days of surgery completed oral antibiotic treatment at home	1000 mg orally every 8 h for 5 days immediately after surgery
**Follow-up**		30 days after cholecystectomy	30 days postoperatively	4 weeks postoperatively	30 days postoperatively	30 days after cholecystectomy	Four weeks postoperatively	30 postoperative days
**Diagnosis of acute cholecystitis**		The diagnosis AC was established according to the Tokyo Guidelines 07	Clinical and radiological signs of acute cholecystitis grades I and II		The diagnosis of AC was based on the Tokyo Guidelines 13	AC was defined according to the Tokyo Guidelines	AC was defined according to the Tokyo Guidelines	Diagnosis of mild or moderate ACC according to the Revised Tokyo Guidelines
**Description of cholecystectomy**		LC using the four-trocar technique according to the guidelines of the Dutch Society of Surgery, which included establishing the critical view of safety	LC, but four patients were included based on the primary intent to perform laparoscopic cholecystectomy, but the surgeon responsible for the procedure decided to do an open procedure for technical reasons	In most cases, LC was performed by the three-trocar technique. A fourth trocar was additionally inserted in special cases. All the operations were performed by LC-specialized surgeons who had performed more than 1000 cases	In most cases, LC was done using the standard technique with three trocars. A fourth trocar was additionally inserted in special cases	LC was performed by the four-trocar technique, with transection of the cystic duct and artery after reaching the critical view of safety as described by Strasberg	The surgical approach (laparoscopic or open cholecystectomy), intraoperative cholangiography, and abdominal drainage were performed according to each surgeon’s preferences and standard practice	The American technique for LC was used, and intraoperative cholangiography was used as a routine in all patients after having achieved the “critical view of safety”

*Abbreviations*: *ACC* acute calculous cholecystitis, *AC* acute cholecystitis, *LC* laparoscopic cholecystectomy, *ERCP* endoscopic retrograde cholangiopancreatography, *AIDS* acquired immunodeficiency syndrome

**Table 3:** Baseline characteristics of the included studies

Study ID	Sample *n* (%)			Age, year, mean (SD)		Sex, female *n* (%)		BMI, kg/m^2^, mean (SD)	
	**Antibiotic**	**Placebo**	**Total**	**Antibiotic**	**Placebo**	**Antibiotic**	**Placebo**	**Antibiotic**	**Placebo**
Braak et al. 2022	226	231	457	58.0 (13.9)	57.5 (14.6)	119 (52.7)	114 (49.4)	28.8 (5.2)	28.7 (5.1)
Jaafar et al. 2020^a^	42	48	90	48.5 (24)	49 (25)	24(57.1)	25(52.1)	27 (7)	28 (6)
Park et al. 2023	125 (50.6)	122 (49.4)	247	51.6 (15.51)	52.4(13.71)	62 (49.6)	75 (61.5)	25 (3.47)	24.5 (3.79)
Kim et al. 2017	93	95	188	52.1 (15.3)	52(15)	44(47.31)	49(51.58)	24.8 (3.4)	25 (4)
Loozen et al. 2017^a^	77	73	150	52 (66)	54 (58)	45 (58.4)	35 (48)	-	-
Regimbeau et al. 2014^a^	207	207	414	55 (75)	56 (74)	107(51.7)	103(49.8)	-	-
Santibañes et al. 2018	96	105	201	49.9 (14.7)	49.9 (14.3)	44(45.8)	57(54.3)	28.6 (5.2)	28.2 (4.3)

^a^Data are presented as median and interquartile

### Risk of bias assessment results

**Fig. 2:**
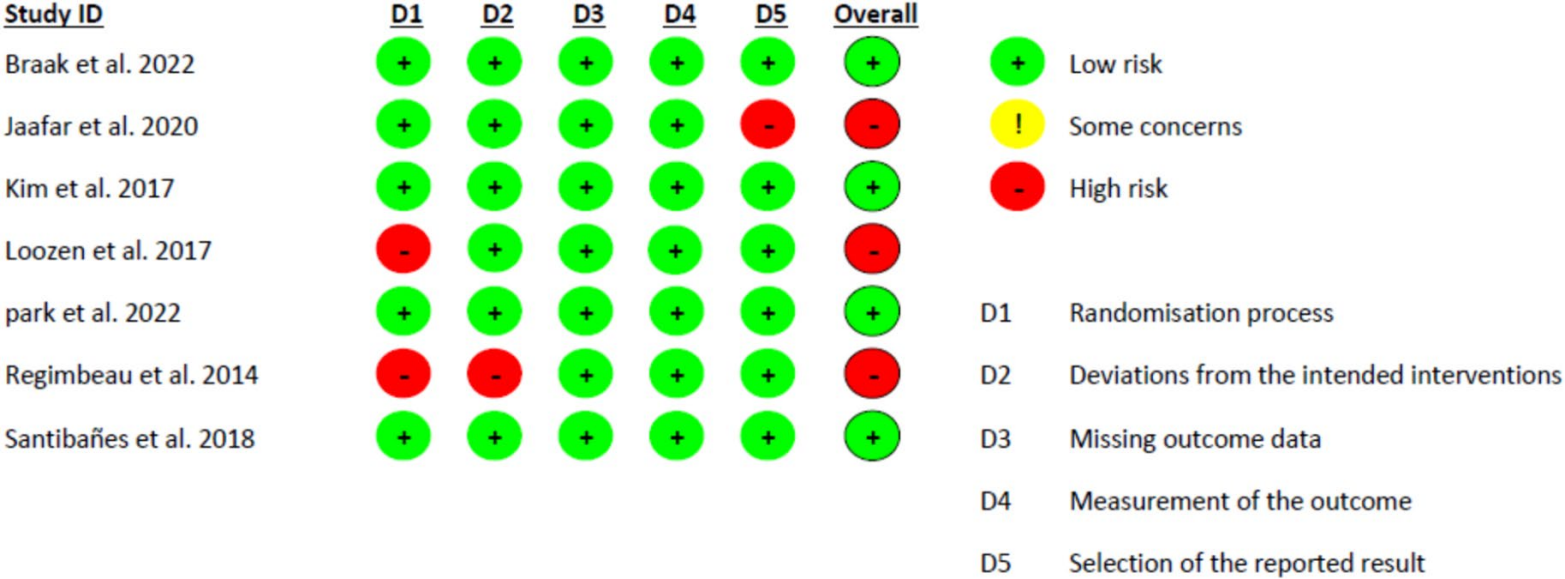
Risk of bias assessment of the included studies according to Cochrane risk of bias assessment tool 2

According to ROB2 (2023), four studies showed a low risk of bias (Braak et al. 2022; Park et al. 2023; Kim et al. 2017; Santibañes et al. 2018), while three showed a high risk (Jaafar et al. 2020; Regimbeau et al. 2014; Loozen et al. 2017). Jaffar et al. (2020) did not report some of the secondary outcomes as planned in their protocol, which introduces a significant risk of reporting bias. Loozen et al. (2017) had concerns regarding the randomization process; neither the patients nor the investigators were blinded to the allocation process. Furthermore, the study conducted by Regimbeau et al. (2014) revealed a higher proportion of patients with diabetes mellitus in the treatment group (27%) compared to the control group (13%). This discrepancy raises concerns regarding potential biased allocation and divergence from the planned study arm. The quality assessment of the included studies is shown in Fig. 2.

### Postoperative infectious complications

**Fig. 3:**
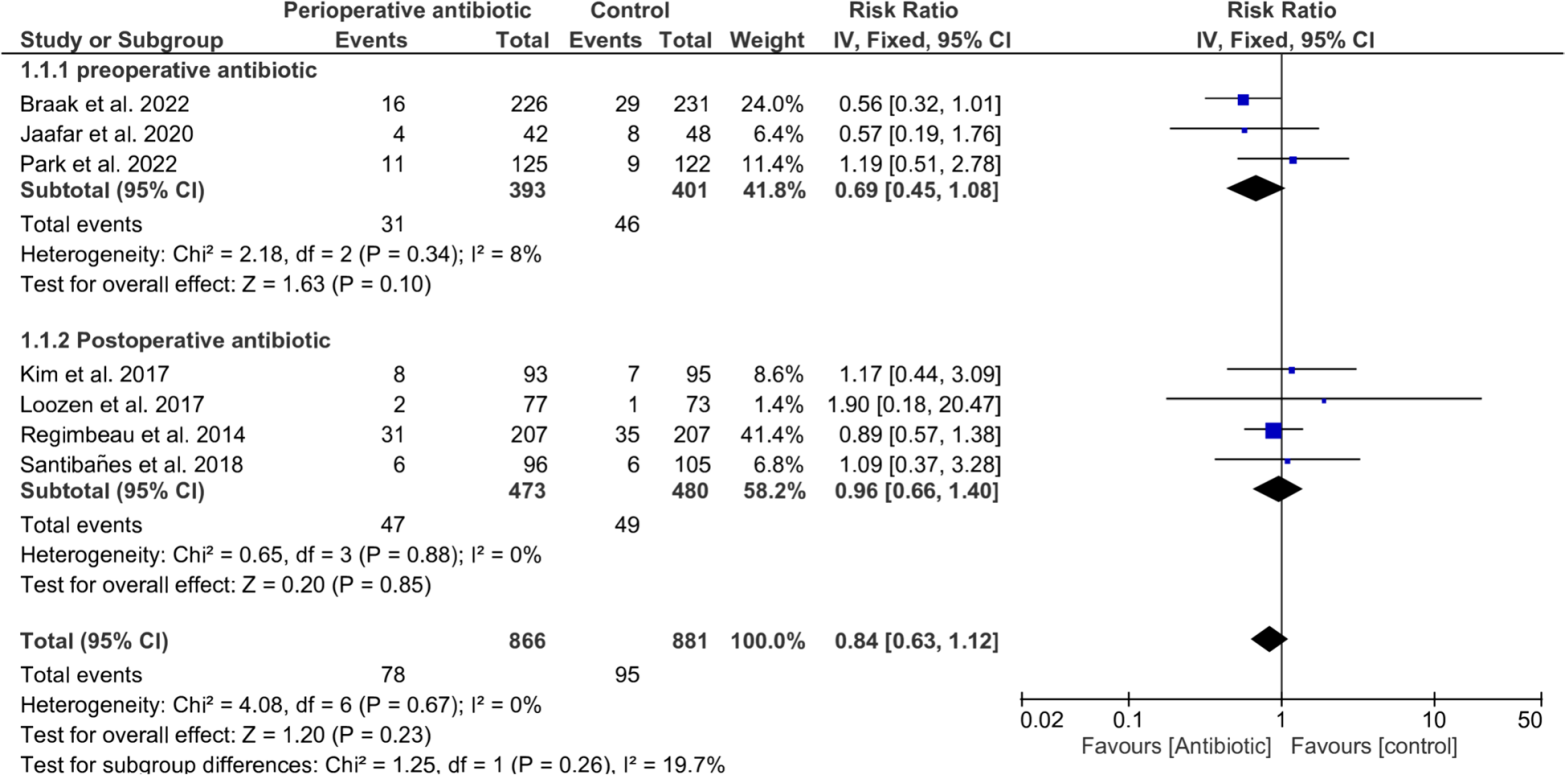
Forest plot of total postoperative infectious complications

Our analysis resulted in no significant difference regarding total PIC (RR = 0.84 with 95% CI (0.63, 1.12), *P* = 0.23) (*I*^2^ = 0%, *P* = 0.67), preoperatively administered antibiotics (RR = 0.69 with 95% CI (0.45, 1.08), *P* = 0.10) (*I*^2^ = 8%, *P* = 0.34), and postoperatively administered antibiotics (RR = 0.96 with 95% CI (0.66, 1.40), *P* = 0.85) (*I*^2^ = 0%, *P* = 0.88), as shown in Fig. 3.

### Surgical site infection

**Fig. 4:**
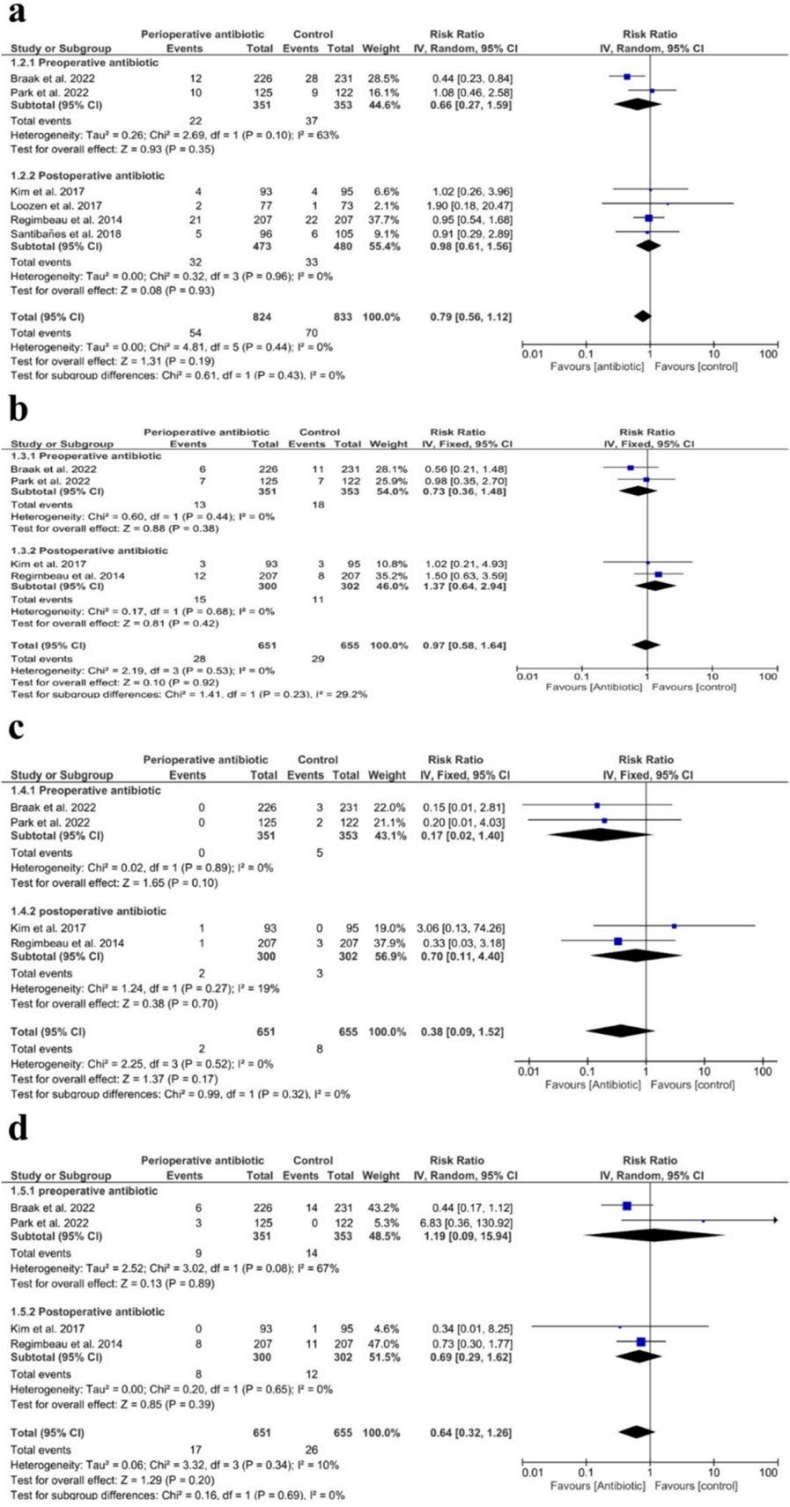
Forest plot of surgical site infections. **a** Total surgical site infections; **b** superficial surgical site infection; **c** deep surgical site infection; **d** organ and/or space surgical site infection

There were no significant differences regarding total SSI (RR = 0.79 with 95% CI (0.56, 1.12), *P* = 0.19) (*I*^2^ = 0%, *P* = 0.44), preoperatively administered antibiotics (RR = 0.66 with 95% CI (0.27, 1.59), *P* = 0.06) (*I*^2^ = 63%, *P* = 0.10), and postoperatively administered antibiotics (RR = 0.98 with 95% CI (0.61, 1.56), *P* = 0.23) (*I*^2^ = 0%, *P* = 0.96), as shown in Fig. 4A*.* Also, there were no significant differences in each type of SSI: superficial SSI (RR = 0.97 with 95% CI (0.58, 1.64), *P* = 0.92) (*I*^2^ = 0%, *P* = 0.53), preoperatively administered antibiotics (RR = 0.73 with 95% CI (0.36, 1.48,* P* = 0.38) (*I*^2^ = 0%, *P* = 0.44), and postoperatively administered antibiotics (RR = 1.37 with 95% CI (0.64, 2.94), *P* = 0.42) (*I*^2^ = 0%, *P* = 0.68), as shown in Fig. 4B; deep SSI (RR = 0.38 with 95% CI (0.09, 1.52), *P* = 0.17) (*I*^2^ = 0%, *P* = 0.52), preoperatively administered antibiotics (RR = 0.17 with 95% CI (0.02, 1.40), *P* = 0.10) (*I*^2^ = 0%, *P* = 0.89), and postoperatively administered antibiotics (RR = 0.70 with 95% CI (0.11, 4.40), *P* = 0.70) (*I*^2^ = 19%, *P* = 0.27), as shown in Fig. 4C; organ and/or space SSI (RR = 0.64 with 95% CI (0.32, 1.26), *P* = 0.20) (*I*^2^ = 10%, *P* = 0.34), preoperatively administered antibiotics (RR = 1.19 with 95% CI (0.09, 15.94), *P* = 0.89) (*I*^2^ = 67%, *P* = 0.08), and postoperatively administered antibiotics (RR = 0.69 with 95% CI (0.29, 1.62), *P* = 0.39) (*I*^2^ = 0%, *P* = 0.65), as shown in Fig. 4D.

### Distant infections

**Fig. 5:**
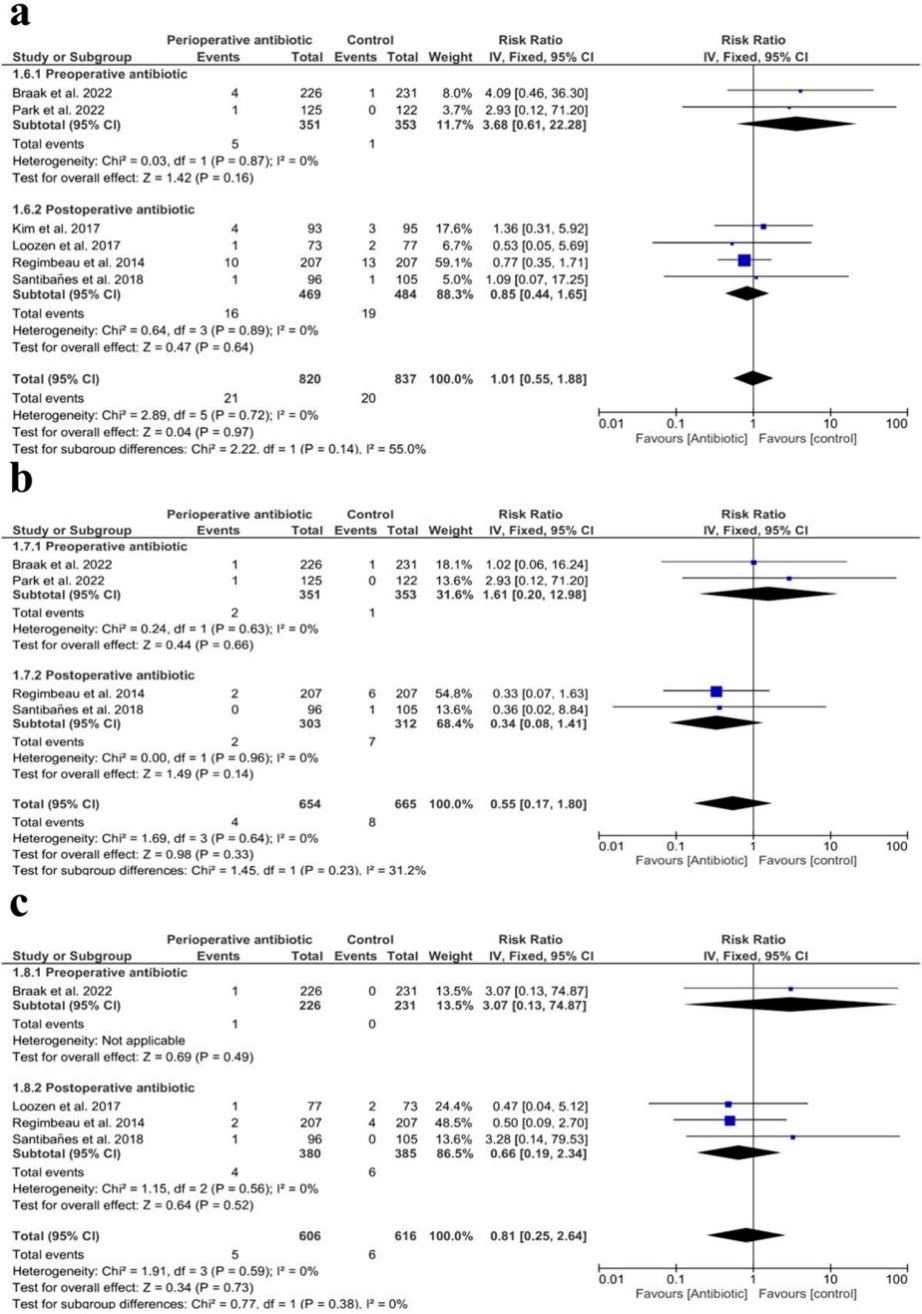
Forest plot of postoperative distant infections. **a** Total number of postoperative distant infections; **b** pneumonia; **c** urinary tract infection

We found no significant difference in the total number of postoperative distant infections (RR = 1.01 with 95% CI (0.55, 1.88), *P* = 0.97) (*I*^2^ = 0%, *P* = 0.72) (*I*^2^ = 0%, *P* = 0.72), preoperatively administered antibiotics (RR = 3.68 with 95% CI (0.61, 22.28), *P* = 0.16) (*I*^2^ = 0%, *P* = 0.87), and postoperatively administered antibiotics (RR = 0.85 with 95% CI (0.44, 1.65), *P* = 0.64) (*I*^2^ = 0%, *P* = 0.89), as shown in Fig. 5A*.* Similarly, there were no significant differences in pneumonia (RR = 0.55 with 95% CI (0.17, 1.80), *P* = 0.33) (*I*^2^ = 0%, *P* = 0.64), preoperatively administered antibiotics (RR = 1.61 with 95% CI (0.20, 12.98), *P* = 0.66) (*I*^2^ = 0%, *P* = 0.63), and postoperatively administered antibiotics (RR = 0.34 with 95% CI (0.08, 1.41), *P* = 0.14) (*I*^2^ = 0%, *P* = 0.96), as shown in Fig. 5B. Our analysis did not show any significant difference in UTI (RR = 0.81 with 95% CI (0.25, 2.64), *P* = 0.73) (*I*^2^ = 0%, *P* = 0.59), preoperatively administered antibiotics (RR = 3.07 with 95% CI (0.13, 74.87), *P* = 0.49) and postoperatively administered antibiotics (RR = 0.66 with 95% CI (0.19, 2.34), *P* = 0.52) (*I*^2^ = 0%, *P* = 0.56), as shown in Fig. 5C*.*

### Non-infectious complications

**Fig. 6:**
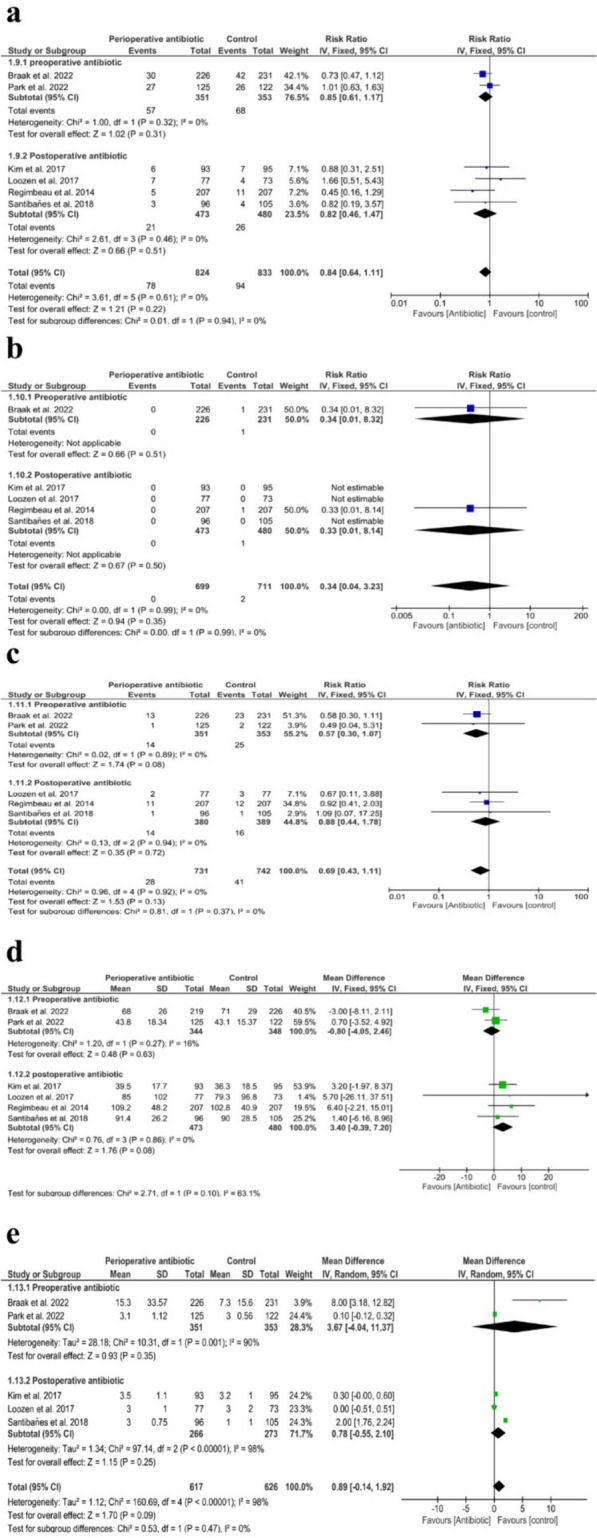
Forest plots of noninfectious morbidity and mortality. **a** The overall incidence of postoperative non-infectious complications; **b** mortality; **c** readmission; **d** operation time (minutes); **e** length of hospital stay (days)

Our analysis indicated that there were no significant differences in the overall incidence of postoperative non-infectious complications (RR = 0.84 with 95% CI (0.64, 1.11), *P* = 0.22) (*I*^2^ = 0%, *P* = 0.61), preoperatively administered antibiotics (RR = 0.85 with 95% CI (0.61, 1.17), *P* = 0.31) (*I*^2^ = 0%, *P* = 0.32), and postoperatively administered antibiotics (RR = 0.82 with 95% CI (0.46, 1.47), *P* = 0.51), (*I*^2^ = 0%, *P* = 0.46), as shown in Fig. 6A.

We found no significant difference in mortality (RR = 0.34 with 95% CI (0.04, 3.23), *P* = 0.35) (*I*^2^ = 0%, *P* = 0.99), preoperatively administered antibiotics (RR = 0.34 with 95% CI (0.01, 8.32), *P* = 0.51), and postoperatively administered antibiotics (RR = 0.33 with 95% CI (0.01, 8.14), *P* = 0.50), as shown in Fig. 6B*.*

The pooled result of the meta-analysis showed comparable readmission rates in both groups (RR = 0.69 with 95% CI (0.43, 1.11), *P* = 0.13) (*I*^2^ = 0%, *P* = 0.92), preoperatively administered antibiotics (RR = 0.57 with 95% CI (0.30, 1.07), *P* = 0.08) (*I*^2^ = 0%, *P* = 0.89), and postoperatively administered antibiotics (RR = 0.88 with 95% CI (0.44, 1.78), *P* = 0.72) (*I*^2^ = 0%, *P* = 0.94), as shown in Fig. 6C*.*

Operation time showed no change with either group (MD = 0.98 min with 95% CI (-1.49, 3.45), *P* = 0.44) (*I*^2^ = 0%, *P* = 0.46), preoperatively administered antibiotics (MD = -0.80 min with 95% CI (-4.05, 2.46), *P* = 0.63) (*I*^2^ = 16%, *P* = 0.27), and postoperatively administered antibiotics (MD = 3.40 min with 95% CI (-0.39, 7.20), *P* = 0.08) (*I*^2^ = 0%, *P* = 0.86), as shown in Fig. 6D*.*

Our results showed that the length of hospital stay was equal in both groups (MD = 0.89 day with 95% CI (-0.14, 1.92), *P* = 0.09) (*I*^2^ = 98%, *P* < 0.00001), preoperatively administered antibiotics (MD = 3.67 day with 95% CI (-4.04, 11.37), *P* = 0.35) (*I*^2^ = 90%, *P* = 0.001), and postoperatively administered antibiotics subgroup (MD = 0.78 day with 95% CI (-0.55, 2.10), *P* = 0.25) (*I*^2^ = 98%, *P* < 0.00001), as shown in Fig. 6E*.* Heterogeneity within the postoperatively administered antibiotics were addressed when excluding results of Santibanes et al. (2018) (*I*^2^ = 0%, *P* = 0.32), and the results did not change in this subgroup (MD = 0.21 day with 95% CI (-0.22, 0.48), *P* = 0.09).

## Discussion

In this systematic review and meta-analysis, we aimed to assess the efficacy of perioperative antibiotic administration in reducing PIC in patients with AC undergoing emergency cholecystectomy. We found no significant difference in total PIC, SSI, or any of its components separately (superficial, deep, and organ or space SSI), distant infections or any of its components (pneumonia and UTI), non-infectious complications, mortality, hospital readmission, and operation time either with perioperative antibiotic or with no antibiotic. Additionally, there was no difference in outcomes based on whether antibiotics were administered before or after surgery. The results of the pooled studies were homogenous in nearly all outcomes, which reflects the agreement of pooled results.

Braak et al. (2022), Park et al. (2023), Jaafar et al. (2020), Kim et al. (2017), Loozen et al. (2017), Regimbeau et al. (2014), and de Santibañes et al. (2018) found in their RCTs that there is no significant difference between the antibiotic and control groups regarding PIC. Choudhary et al. (2008) reported in their meta-analysis that there is no significant difference in total infection risk between the antibiotic and control group after emergency cholecystectomy.

Regarding SSI, our results indicated that there is no statistically significant difference between the antibiotics and control groups. These results are in line with the RCTs of Jaafar et al. (2020), Kim et al. (2017), Loozen et al. (2017), Regimbeau et al. (2014), and de Santibañes et al. (2018). Furthermore, Hajibandeh et al. (2019) conducted a meta-analysis of four RCTs to assess the effectiveness of antibiotics in reducing postoperative SSI and found no association. La Regina and colleagues (2019) reported in their meta-analyses of three RCTs that postoperative antibiotics do not reduce SSI. However, Braak et al. (2022) reported that SSI may have a higher predominance among the control group. It should be noted that the control group in Braak et al. (2022) had a higher white blood cell count upon admission, which could lead to biased observation.

In terms of distant infections, we found that there is no statistically significant difference between the antibiotic and control groups. Also, our results are aligned with Braak et al. (2022), Choudhary et al. (2008), and Hajibandeh et al. (2019) regarding postoperative distant infections.

Although up to 20% of patients with AC may experience bacterial infection due to cystic duct obstruction and bile stasis, AC is still primarily an inflammatory process, and that may explain why antibiotic administration did not lead to lower rates of infection in our study or the literature van Dijk (2016). Moreover, in patients with positive bile culture, antibiotic treatment does not always prevent complications in patients with AC (Galili et al. 2008). Despite the 2018 Tokyo guidelines (Gomi et al. 2018) recommending the use of preoperative and intraoperative antibiotics for uncomplicated cholecystitis patients, the current study and existing literature suggest that such antibiotics do not provide protective benefits against infections. This raises concerns about the routine use of antibiotics in such procedures and calls for a reevaluation of current practices. Given these findings, it is crucial to adopt a cautious and selective approach to antibiotic use, particularly considering the growing challenge of antibiotic resistance (Llor and Bjerrum 2014). Instead, we recommend focusing on enhancing surgical techniques and providing quality postoperative care. These measures aim to improve patient outcomes while reducing the need for antibiotics.

Our research has significant implications for the economy, potentially reducing hospital stays and antibiotic expenses, easing the burden on healthcare systems, and preventing future antibiotic resistance. This supports the aims and goals of antibiotic stewardship programs (ASPs) (Karanika et al. 2016). It highlights the need for a thoughtful approach to antibiotic use in cholecystectomy procedures. These insights are important not only for medical professionals but also for healthcare quality improvement and sustainability researchers.

On the other hand, Yang et al. (2021) conducted a meta-analysis on patients with mild to moderate cholecystitis undergoing elective LC and reported that the administration of perioperative antibiotics could effectively reduce infections, including SSI and distant infections. The reason why antibiotics show efficacy with patients undergoing elective LC in the study of Yang et al. (2021) but not here in our study on patients undergoing emergency cholecystectomy is a very interesting question. One reason could be credited to the different pathologies between acute and chronic cholecystectomy. In contrast, a long period of bile stasis in chronic cholecystitis can predispose to organism growth; the relatively short period of AC is not always associated with colonization or bacteriobilia. We hypnotize that this different outcome may be attributed to a distinct feature in Yang et al. (2021). They included 14 RCTs, and of them, 6 (43%), including 2573 patients (59% of the meta-analysis sample size), were conducted in Asia, and a subgroup analysis found the antibiotics are effective in reducing total infections (*P* = 0.003), SSI (*P* = 0.006), and distant infections (*P* = 0.005) only in studies from Asia, but not from Europe or America. The reason why antibiotics are effective in Asian patients is yet to be studied.

Interestingly, our investigation revealed a notable finding: The utilization of antibiotics was associated with a 25% increase in the duration of hospital stays. This finding represents a good example of ASPs, which aim to improve antimicrobial use to improve patient outcomes, reduce antibiotic costs, and minimize the side effects associated with antimicrobial use, including drug resistance. Additionally, certain cases might exhibit hypersensitivity reactions to specific antibiotics, necessitating an extended stay for closer observation.

Furthermore, our study’s other outcomes showed no statistically significant differences concerning readmission rates, occurrences of non-infectious complications, and the duration of the surgical procedure. These results align with the findings from Hajibandeh et al. (2019).

### Strengths

We are reporting a very important example of antimicrobial overuse with no obvious benefits in patients undergoing emergency cholecystectomy. We included seven RCTs, and their pooled results were homogenous, which robustness the agreement on the uselessness of antimicrobial treatment. We did a subgroup analysis depending on the time of antibiotic administration, and we found similar results, which was a limitation of a previous meta-analysis (Hajibandeh et al. 2019).

### Limitations

However, it is essential to acknowledge the limitations inherent in our study. Specifically, three of the included RCTs are potentially susceptible to bias. The previous bias might influence the robustness of our conclusions. Our meta-analysis only included RCTs published in English, potentially excluding relevant studies published in other languages. Also, some studies used different antibiotic regimens. Braak et al. (2022) and Loozen et al. (2017) used 2 g of first-generation cephalosporin; Jaafar et al. (2020) used 4 g of piperacillin/tazobactam, and Kim et al. (2017) used 1.0 g of second-generation cephalosporin. While Park et al. (2023) used 1.0 g of first-generation cephalosporin, Regimbeau et al. (2014) used an amoxicillin regimen, and Santibañes et al. (2018) used an ampicillin/sulbactam regimen. These limitations may impact the overall comprehensiveness of our meta-analysis and underscore the necessity for cautious interpretation and consideration when evaluating the scope and applicability of our results. There were no studies that reported on the occurrence of antibiotic-associated (pseudomembranous) colitis caused by *Clostridium difficile*. We were unable to conduct a subgroup analysis on the severity of AC as there were no sufficient data available. Two of the included studies (Jaafar et al. 2020; Regimbeau et al. 2014) included patients undergoing open cholecystectomy, which may introduce a confounding variable; however, the percentage was very small.

## Conclusion

The current evidence on the administration of prophylactic perioperative antibiotics in patients with mild to moderate acute cholecystitis did not show a significant reduction of postoperative infectious complications after emergency cholecystectomy. This meta-analysis recommends revising the current guidelines on the use of antibiotics in acute cholecystitis, especially with the growing challenges of antimicrobial resistance.

### Supplementary Information

Supplementary  Material 1: Supplementary Table 1. Search strategy for each database. 

## Data Availability

All raw data presented or analyzed in this article are available on request from the corresponding author. Competing interests The authors declare no competing interests.

## Abbreviations

AC: Acute cholecystitis
PIC: Postoperative infectious complications
RCTs: Randomized controlled trials
AMSTAR-2: Assessing the Methodological Quality of Systematic Reviews 2
PRISMA: Preferred Reporting Items for Systematic Reviews and Meta-Analyses
PROSPERO: International Prospective Register of Systematic Reviews
WOS: Web of Science
ROB2: Cochrane Collaboration Risk of Bias Assessment Tool 2
BMI: Body mass index
SSI: Surgical site infection
UTI: Urinary tract infection
RR: Risk ratio
CI: Confidence interval
MD: Mean difference
GRADE: Recommendations Assessment, Development, and Evaluation of evidence
LC: Laparoscopic cholecystectomy
ASPs: Antibiotic stewardship programs
