# Supplementary material for: Antibiotic prophylaxis in emergency cholecystectomy for mild to moderate acute cholecystitis: a systematic review and meta-analysis of randomized controlled trials
Source: Perioper Med (Lond). 2024 Aug 9;13:87. doi: 10.1186/s13741-024-00441-4 (PMC11312388; doi:10.1186/s13741-024-00441-4)
Supplement: Supplementary file 1 — Supplementary Material 1: Supplementary Table 1. Search strategy for each database. [file 13741_2024_441_MOESM1_ESM.docx]

| **Supplementary Table 1.** Search strategy for each database | |
| --- | --- |
| **Database** | Search strategy |
| **PubMed** | (cholecystectomy) AND ((Acute) OR (Emergency)) AND (antibiotic*) |
| **Embase** | ('cholecystectomy'/exp OR cholecystectomy) AND (acute OR 'emergency'/exp OR emergency) AND antibiotic* |
| **Cochrane CENTRAL** | (cholecystectomy) AND ((Acute) OR (Emergency)) AND (antibiotic*) |
| **WOS** | (cholecystectomy) AND ((Acute) OR (Emergency)) AND (antibiotic*) |
| **Scopus** | TITLE-ABS-KEY ( ( cholecystectomy )  AND  ( ( acute )  OR  ( emergency ) )  AND  ( antibiotic* ) ) |
